# Regulatory T cells limit age-associated retinal inflammation and neurodegeneration

**DOI:** 10.1186/s13024-024-00724-w

**Published:** 2024-04-05

**Authors:** María Llorián-Salvador, Alerie G. de la Fuente, Christopher E. McMurran, Amy Dashwood, James Dooley, Adrian Liston, Rosana Penalva, Yvonne Dombrowski, Alan W. Stitt, Denise C. Fitzgerald

**Affiliations:** 1 Wellcome-Wolfson Institute for Experimental Medicine, Queen’s University Belfast, Belfast, Northern Ireland BT9 7BL UK; 2 Vall d’Hebron Research Institute (VHIR), Universitat Autónoma de Barcelona, 08035 Barcelona, Spain; 3 Institute for Health and Biomedical Research of Alicante (ISABIAL) Alicante, 03010 Alicante, Spain; 4 Instituto de Neurociencias CSIC-UMH, San Juan de Alicante, 03550 Alicante, Spain; 5 Department of Clinical Neurosciences, University of Cambridge, Cambridge, UK; 6 Department of Medical Epidemiology and Biostatistics, Karolinska Institute, Stockholm, Sweden; 7 Babraham Institute, Cambridge, UK; 8 Department of Pathology, University of Cambridge, Cambridge, UK

**Keywords:** Ageing, Inflammation, Neurodegeneration, Regulatory T cells, Retina

## Abstract

**Background:**

Ageing is the principal risk factor for retinal degenerative diseases, which are the commonest cause of blindness in the developed countries. These conditions include age-related macular degeneration or diabetic retinopathy. Regulatory T cells play a vital role in immunoregulation of the nervous system by limiting inflammation and tissue damage in health and disease. Because the retina was long-considered an immunoprivileged site, the precise contribution of regulatory T cells in retinal homeostasis and in age-related retinal diseases remains unknown.

**Methods:**

Regulatory T cells were selectively depleted in both young (2–4 months) and aged (18–23 months) FoxP3-DTR mice. We evaluated neuroretinal degeneration, gliosis, subretinal space phagocyte infiltration, and retinal pigmented epithelium morphology through immunofluorescence analysis. Subsequently, aged Treg depleted animals underwent adoptive transfer of both young and aged regulatory T cells from wild-type mice, and the resulting impact on neurodegeneration was assessed. Statistical analyses employed included the U-Mann Whitney test, and for comparisons involving more than two groups, 1-way ANOVA analysis followed by Bonferroni’s post hoc test.

**Results:**

Our study shows that regulatory T cell elimination leads to retinal pigment epithelium cell dysmorphology and accumulation of phagocytes in the subretinal space of young and aged mice. However, only aged mice experience retinal neurodegeneration and gliosis. Surprisingly, adoptive transfer of young but not aged regulatory T cells reverse these changes.

**Conclusion:**

Our findings demonstrate an essential role for regulatory T cells in maintaining age retinal homeostasis and preventing age-related neurodegeneration. This previously undescribed role of regulatory T cells in limiting retinal inflammation, RPE/choroid epithelium damage and subsequently photoreceptor loss with age, opens novel avenues to explore regulatory T cell neuroprotective and anti-inflammatory properties as potential therapeutic approaches for age-related retinal diseases.

**Supplementary Information:**

The online version contains supplementary material available at 10.1186/s13024-024-00724-w.

## Synopsis for graphical abstract

Regulatory T cells (Treg) have an important role in maintaining a healthy retina, particulary in aged mice.

- Eliminating Treg leads to inflammation and neurodegeneration in the retina of aged but not young mice.
- Absence of Treg leads to the accumulation of inflammatory cells in the subretinal space and disturbance of the retinal pigment epithelium barrier in both young and aged mice.
- Adoptive transfer of young but not aged Treg partially rescued aged mice from retinal neurodegeneration.

## Background

Ageing represents the most significant risk factor for various sight-threatening retinal diseases, such as diabetic retinopathy, age-related macular degeneration (AMD) or glaucoma. Although the precise pathobiology underpinning age-related retinal degeneration remains ill-defined, there is broad recognition of the crucial role played by chronic low-grade chronic inflammation [1, 2]. Historically, the retina has long been regarded as immunoprivileged. This privileged status is maintained by protective retinal-blood-barriers such as the retinal pigment epithelium (RPE) and a localized immune system including microglia, which prevent the entry of pathogens and systemic inflammation into the retina [3]. The ageing retina is characterized by chronic low-grade inflammation (also referred to as “para-inflammation” or “inflammaging”) resulting in age-mediated endogenous insults and impaired defense mechanisms (i.e. reduced phagocytic activity, migration from microglia, disruption of blood-retinal barrier, etc.). However, it is now recognised that age-related immune dysregulation contributes to retinal degeneration even in the absence of overt disease, challenging the notion of complete immune privilege in the retina. This dysregulation leads to detrimental age-related pathologies such as AMD, diabetic retinopathy or glaucomatous retinopathy [3, 4].

Regulatory T cells (Treg) maintain and restore tissue homeostasis by regulating immune responses and inflammation and promoting tissue regeneration in a range of tissues [5]. In recent years, an expanded exploration of Tregs has revealed novel functionalities beyond conventional immune regulatory roles. Interest has increased in understanding Treg regenerative and neuroprotective properties, particularly within the central nervous system (CNS). In the CNS, Tregs have key neuroprotective functions by modulating microglial and astrocyte activation to prevent gliosis and inflammation in ischemic stroke, but also play a crucial role enhancing remyelination to limit axonal degeneration in multiple sclerosis models [6–8]. In other neurodegenerative disorders like Alzheimer's and Parkinson's disease, Tregs have demonstrated effectiveness in restoring homeostasis and preventing cognitive decline. This involves eliminating neuroimmunogens, polarizing microglia, modulating astrocytes, and restoring normal neuronal function [9, 10]. Furthermore, interventions aimed at enhancing astrocyte-mediated IL-2 production to expand local Treg populations in the CNS have shown promise in mitigating inflammatory damage and facilitating repair in mouse models of traumatic brain injury, stroke, multiple sclerosis and age-associated cognitive decline [11, 12].

In the healthy retina, Treg density is very low, although Treg infiltration in the neuropil occurs during retinal inflammatory diseases and retinal pathology [13–15]. During acute retinal inflammation, such as in uveitis, Treg exert immunosuppressive functions which counterbalance uncontrolled immune responses and thereby limit degenerative pathology [14]. Ischemia also increases Treg density in the retina where they participate in preventing supra-retinal damage and fostering reparative intra-retinal angiogenesis [15], demonstrating that Treg play important roles in retinal disease. Yet the role of Treg in age-associated retinal pathology has been largely unexplored.

As we age, Treg cells exhibit a higher frequency or increased activity and function [16, 17], but whether this supports immune regulation in the aged retina is unknown. To address this, we asked whether genetic ablation of Treg impairs retinal homeostasis in young and aged mice. Here, we show that Treg depletion leads to accelerated retinal neurodegeneration and gliosis in aged but not young mice. Thus, we demonstrate that Treg have a pivotal role in preventing aged retinal neurodegeneration and subsequent gliosis. We have also shown that young Treg are more efficient than aged Treg in reversing age-related retinal degeneration. This work sets the groundwork to investigate Treg immunosuppressive and neuroprotective capacity as an effective treatment for age related retinal degenerative diseases.

## Methods

### Animals

All animals were housed and bred in a standard pathogen free experimental facility and exposed to a 12 h light/dark cycle with free access to food and water. All mice were bred in-house or purchased from Charles River Laboratories, UK. All procedures were conducted under the regulation of the UK Home Office Animals (Scientific Procedures) Act 1986. This study was approved by the Animal Welfare and Ethical Review body (AWERB) of Queen's University Belfast and ethical review committee and all animal maintenance, and experiments were done in according with the UK Home Office Regulation (Project Licenses 2789 and 2894). B6.129(Cg)-Foxp3^tm3(Hebgf/GFP)Ayr^/J (FoxP3-DTR) mice were kindly provided by Prof. Alexander Rudensky (Memorial Sloan Kettering Institute, New York) and Dr. Rebecca Ingram (Queen’s University Belfast) and bred on a C57BL6/J background. Following the 3Rs principle and to reduce animal use, the eyes analysed in this project were obtained from animals that underwent demyelination surgery exclusively in the spinal cord as described previously [8]. In brief, all mice underwent 30 min of isofluorane-based anaesthesia to allow spinal cord surgery, during which the eyes were covered with eye drops to prevent dehydration. Mice received a single injection of 1.2µl of 1% (w/v) L L-α-Lysophosphatidylcholine (Lysolecithin; Sigma-Aldrich) into the thoracic spinal cord under general anaesthesia. At day 18 post first DT injection (or day 14 post-surgery), mice were terminally anaesthetised with intraperitoneal (i.p.) pentobarbital injections and transcardially perfused with ice-cold phosphate buffered saline (PBS) followed by 4% paraformaldehyde (PFA) (Sigma-Aldrich). Eyes were removed and immersed in 4% PFA overnight at 4 °C. Then, eyes reserved for cryosectioning were cryoprotected with 30% sucrose in PBS for 72h, snap-frozen in OCT (Tissue-Tek), cryosectioned at 15 µm thickness and immunostained as indicated below. Eyes that were used for flatmount staining were fixed overnight and transferred to PBS.

### Treg depletion

Endogenous Treg were depleted from young (2–4 months) and aged (18–23 months) male and female Foxp3-DTR mice using diphtheria toxin (DT). Mice received daily i.p. injections of DT (0.04 µg/g of body weight; Sigma, Cat. No. D0564) for 3 consecutive days. Young (2-4m) and aged (16m) male and female C57BL6/J were used as controls for DT-associated side effects. To maintain endogenous Treg depletion throughout the course of the study, all mice received an i.p. DT injection (0.04µg/g of body weight) every fourth day up until day 18, when mice were sacrificed. Control animals received 200 µl of saline i.p. Depletion was confirmed at the endpoint by flow cytometric analysis of Foxp3 expression in blood, spleen, and lymph nodes.

### Natural Treg isolation and adoptive transfer

Young (2–4 months) and aged (15–18 months) female C57BL6J mice were culled by CO_2_ overdose. Lymph nodes and spleens were dissected and mashed into a single cell suspension using a 5ml syringe plunger. Single cell suspension was passed through a 70 µm strainer and then subjected to CD4 negative and CD25 positive immunomagnetic selection following manufacturer’s instructions. In brief, cells were incubated with CD4 negative selection kit (STEMCELL Technologies) for 15 min on ice and CD4^−^ cells were magnetically removed. Then, CD4^+^ cells were subjected to CD25^+^ cell isolation (STEMCELL Technologies). Cells were resuspended at 10^6^ cells per 200µl saline and injected i.p. only once on the same day as the third DT injection (the day prior to spinal cord surgery).

### Flow cytometry

#### Spleen and lymph nodes

To confirm cell purity isolated CD4^+^CD25^+^ Treg were subjected to flow cytometry staining as described below. To confirm Treg depletion spleen and lymph nodes were mashed through a 70 µm strainer and then treated with red blood cell lysis buffer (STEMCELL Technologies) for 2 min at room temperature. Cells obtained from lymph nodes and spleens were then washed with PBS and centrifuged at 300 g for 5 min at 4°C. Cells were resuspended in 200 µL PBS and stained with a cell viability dye with eFluor 506 viability dye (1:2000; ThermoFisher Scientific) and cell surface stained with antibodies for CD4 (1:500; eBioscience, clone RM4.5) and CD25 (1:500; eBioscience, clone PC61.5) for 15 min at RT. Cells were washed with flow cytometry staining buffer (FCSB) (2% FCS in PBS) and centrifuged at 300 g for 5 min at 4 °C. Cells were then fixed with Fix & Perm A (ThermoFisher Scientific) for 10 min at RT. Fixative was washed off with FCSB and centrifuged at 300 g for 5 min at 4 °C. Then, cells were resuspended in 100 µL Fix & Perm B (ThermoFisher Scientific) with an anti-Foxp3 antibody (1:100; eBioscience, clone FJK-16S) overnight at 4 °C. Cells were then washed with FCSB and centrifuged at 300 g and 4 °C for 5 min. Final pellet was resuspended, data were acquired on a FACSCanto II and analyzed using FlowJo software version 9.0 (BD). To calculate cell numbers, singlets were identified by FSC-H versus FSC-A and viable cells gated on CD4 expression, and subsequently CD25^+^ and Foxp3^+^ and CD25^+^Foxp3^+^ cells.

#### Blood

Blood obtained from the animals at the time of perfusion was collected in EDTA-tubes to avoid blood coagulation. 30µl of the blood were incubated with surface antibodies in PBS as indicated per spleen and lymph nodes for 15 min at RT. Cells were washed with flow FCSB and centrifuged at 300 g for 5 min at 4 °C. Red blood cells were then lysed and cells were fixed with Optilyse B (Beckmann and Coulter) for 10 min at RT. Fixative was washed with ddH20 to finish red blood lysis for 15 min and centrifuged at 300 g for 5min at 4 °C. Then, cells were resuspended in 100 µL Fix & Perm B (ThermoFisher Scientific) with an anti-Foxp3 antibody (1:100; eBioscience, clone FJK-16S) overnight at 4 °C. Cells were then washed with FCSB and centrifuged at 300 g and 4 °C for 5 min. Final pellet was resuspended, data were acquired on a FACSCanto II and analyzed using FlowJo software version 9.0 (BD). To calculate cell numbers, singlets were identified by FSC-H versus FSC-A and viable cells gated on CD4 expression, and subsequently CD25^+^ and Foxp3^+^ and CD25^+^Foxp3^+^ cells.

#### Retina and RPE/choroid

Enucleated eyes were microdissected and retina and RPE/choroid separated and conserved in Hibernate A (ThermoFischer Scientific) with 2mM sodium pyruvate. Retina and RPE/choroid were cut in small pieces and subjected to papain digestion for 30 min at 37°C in Hibernate A with 165 units of papain (Worthington) and 40µg/mL DNAse type I (Worthington). Excess of papain was washed-off with Hanks Buffer Salt Solution (HBSS) without calcium or magnesium (ThermoFischer) and cells spun at 300 g for 5 min. Cells were the triturated into a single cell suspension mechanically with a 5 mL pipette and glass polished pipettes in Hibernate A with 4% SOS (Cell Guidance) and 2mM sodium pyruvate (ThermoFischer). Single cell suspension was transferred to a new clean tube through a 70µm strainer and subjected to a 4% BSA gradient for 15 min at 600 g to remove debris and the pigment in the RPE. Cells were resuspended in 250 µL PBS and stained with a cell viability dye with eFluor 506 viability dye (1:2000; ThermoFisher Scientific) and cell surface stained with antibodies for CD4 (1:500; eBioscience, clone RM4.5), CD3 (1:300; eBioscience, clone 145-2c11), CD11b (1:500; eBioscience, Clone M1/70) and CD8 ( 1:500; eBioscience, Clone 53–6.7) for 15 min at RT. Cells were washed with flow cytometry staining buffer (FCSB) (2% FCS in PBS) and centrifuged at 300 g for 5 min at 4 °C. Cells were then fixed with Fix & Perm A (ThermoFisher Scientific) for 10 min at RT. Fixative was washed off with FCSB and centrifuged at 300 g for 5 min at 4 °C. Then, cells were resuspended in 100 µL Fix & Perm B (ThermoFisher Scientific) with an anti-Foxp3 antibody (1:100; eBioscience, clone FJK-16S) and anti-Tbet antibody (1:100; eBioscience, Clone eBio4B10 (4B10)) overnight at 4 °C. Cells were then washed with FCSB and centrifuged at 300 g and 4 °C for 5 min. Final pellet was resuspended, data were acquired on a FACSCanto II and analyzed using FlowJo software version 9.0 (BD). To calculate cell numbers, singlets were identified by FSC-H versus FSC-A and viable cells gated on CD4 expression, and subsequently same number of CD4^+^ T cells were downsampled per animal using FlowJo’s “Downsample” pluging. The downsampled CD4^+^ T cells of each animal were concatenated by age group and then Foxp3 expression and counts were analysed.

### Retinal explants

The method for retinal explants was adapted from a previously published manuscript [18]. Briefly, Retinas from C57BL/6 J mice were placed with the photoreceptor side onto polycarbonate filters (Fisher scientific, UK) in contact with 5% Treg-conditioned media, 5% Treg polarising media or 1µg/mL LPS diluted in DMEM for 6 h at 37 °C. Retinal explants were fixed 1h with 2%PFA.

### Treg conditioned-media generation

Spleens were dissected and mashed through a 70µm strainer into a single cell suspension using a 5ml syringe plunge. Then, red blood cells were lysed using red blood cell lysis (STEMCELL Technologies) for 2min at RT and cells were washed with RPMI media and centrifuged at 300g for 5min at 4 °C. Splenocytes were resuspended in 2% FBS with PBS and rat serum and naïve CD4^+^T cells were magnetically isolated following manufacturer instructions (STEMCELL Technologies). Naïve CD4^+^ T cells were activated with plate bound anti-CD3 (1µg/mL, eBioscience clone 145-2C11) and anti-CD28 in PBS (1µg/mL, eBioscience, clone 37.51). CD4^+^T cells were cultured for 3 days in RPMI 1460 with 10% heat inactivated fetal bovine serum, 1% penicillin/streptomycin, 1% L-Glutamine, 1%HEPES, 1% sodium pyruvate, 1% non-essential amino acids and 50µM 2-mercaptoethanol (all Life Technologies). Naïve CD4^+^T cells were polarized into induced Treg with TGF-β (2ng/mL, R&D Systems), recombinant human IL-2 (10ng/mL, eBioscience) and anti-IFNγ (10µg/mL, clone XMG1.2, Bioxcell). Upon 72h, polarized Treg were collected and centrifuged at 300g for 5min at 4 °C. Treg were then reactivated for another 72h in serum-free X-VIVO L-15 media (Lonza) with Treg polarizing factors as mentioned above. After 72h, conditioned media was harvested, and cell purity checked by flow cytometric staining as indicated above. As control, X-VIVO 15 media with Treg polarizing factors but no cells was harvested upon 72h of incubation.

### Immunostaining

#### Cryosection staining

Eye sections were dried for 30 min at RT and washed in PBS for 10 min. Antigen retrieval was performed using Citrate buffer pH 6.0 (Abcam, ab93678) at 95ºC for 5 min in a water bath and after cooling, and additional 5 min with 10% SDS. Slides were then blocked in 10% Donkey serum (Sigma Aldrich, D9663) in 0.2% Triton-X in PBS for 1h at room temperature. Primary antibodies (Table 1) were added and incubated overnight at 4ºC in 0.5% donkey serum and 0.2% triton-X followed by incubation with secondary antibodies (Table 1) for 1h at room temperature, in PBS.

**Table 1:** Antibodies table

Primary antibody	Cat. No	Company	Host	Dilution
Brn-3a Antibody (C-20)	sc-31984	Santa Cruz Biotechnology	Goat	1:200
Ionized calcium binding adaptor molecule 1 (IBA-1) antibody	ab5076	Abcam	Goat	1:200
Anti-CD68 antibody [FA-11]	ab53444	Abcam	Rat	1:200
Recombinant Anti-PKC alpha antibody [133]	ab11723	Abcam	Mouse	1:500
Anti-Cone Arrestin Antibody	AB15282	Sigma	Rabbit	1:500
Guinea Pig Anti-Synaptophysin	AGP-144	Alomone	Guinea Pig	1:150
Anti-Glial Fibrillary Acidic Protein antibody (GFAP)	Z0334	Agilent	Rabbit	1:500
Anti-Mouse MHC Class II (I-A/I-E) Functional Grade	36–5321-85	Thermofisher	Biotin	1:200
Anti-Secretagogin	RD184120100	Biovendor	Sheep	1:400
DAPI (4′,6-diamidino-2-phenylindole)	D3571	Thermofisher		1:1000
**Secondary antibody**			**Company**	**Cat. No**
Donkey anti-Rabbit IgG (H + L) Highly Cross-Adsorbed Secondary Antibody, Alexa Fluor™ 568			Thermofisher	A10042
Donkey anti-Rabbit IgG (H + L) Highly Cross-Adsorbed Secondary Antibody, Alexa Fluor™ 647			Thermofisher	A-31573
Donkey anti-Rat IgG (H + L) Highly Cross-Adsorbed Secondary Antibody, Alexa Fluor™ 488			Thermofisher	A21208
Donkey anti-Rat IgG (H + L) Highly Cross-Adsorbed Secondary Antibody, Alexa Fluor™ 568			Thermofisher	a11226
Donkey anti-Rat IgG (H + L) Highly Cross-Adsorbed Secondary Antibody, Alexa Fluor™ 647			Thermofisher	A78947
Donkey anti-Mouse IgG (H + L) Highly Cross-Adsorbed Secondary Antibody, Alexa Fluor™ 488			Thermofisher	A21202
Donkey anti-Mouse IgG (H + L) Highly Cross-Adsorbed Secondary Antibody, Alexa Fluor™ 568			Thermofisher	A10037
Donkey anti-Sheep IgG (H + L) Cross-Adsorbed Secondary Antibody, Alexa Fluor™ 488			Thermofisher	A-11015
Donkey anti-Goat IgG (H + L) Cross-Adsorbed Secondary Antibody, Alexa Fluor™ 568			Thermofisher	A11057
Donkey Anti-Goat IgG H&L (Alexa Fluor® 488) (ab150129)			Abcam	ab150129
Donkey Anti-Goat IgG H&L (Alexa Fluor® 647) (ab150131)			Abcam	ab150131
Goat anti-Guinea Pig Alexa Fluor® 568			Abcam	ab175714
Alexa Fluor™ 488 Phalloidin			Thermofisher	A12379

#### Retinal Pigment Epithelium (RPE)/choroid flatmount and retinal explant staining

RPE/choroid flatmounts were dissected under a microdissection microscope (Nikon smz800, Nikon, Tokyo, Japan). The anterior segment of the eye (cornea, lens, iris and ciliary body) was removed, and the retina was carefully peeled off of the RPE/choroidal eyecup.

RPE/choroid flatmounts tissues were washed in PBS followed by treatment with 2% Triton-X 100 for 2h at RT. After washing, samples were blocked with 5% Donkey serum in 0.2% Triton-X 100 for 1 h at RT, and then incubated with the corresponding primary antibody and secondary antibodies. Fixed retinal explants were washed and permeabilised with 2% triton for 3h and incubated with Cone Arrestin antibody for 3h at room temperature and 2h with its secondary antibody. All primary and secondary antibodies used are indicated in Table 1.

#### Image acquisition and analysis

The samples were cover-slipped with Vectashield (H-1000–10, Vector Labs, Burlingame, CA) and examined by Leica DMi8, DM550 epifluorescence microscope or confocal microscope (Leica TCS SP5 and SP8, Leica Microsystems Ltd., Wetzlar, Germany). Further image processing and analysis was performed using Fiji software and blindly manual counting.

### Statistical analysis

Statistical analysis was performed using Graph Pad Prism (GraphPad Software, Inc. version 9 and 10). First, normal distribution was assessed using Kolmogorov–Smirnov tests. When only two groups were compared, such as when checking Treg percentage changes upon Treg depletion U-Mann Whitney was used. For comparisons involving more than two groups, 1-way ANOVA analysis was performed followed by Bonferroni’s multiple comparisons test. When the data was described as percentage, an *arcsin* conversion was performed to analyze the data using parametric tests for normally distributed data. For all statistical tests, differences were considered significant with p values below 0.05.

## Results

### Absence of aged Treg leads to retinal neurodegeneration

**Fig. 1:**
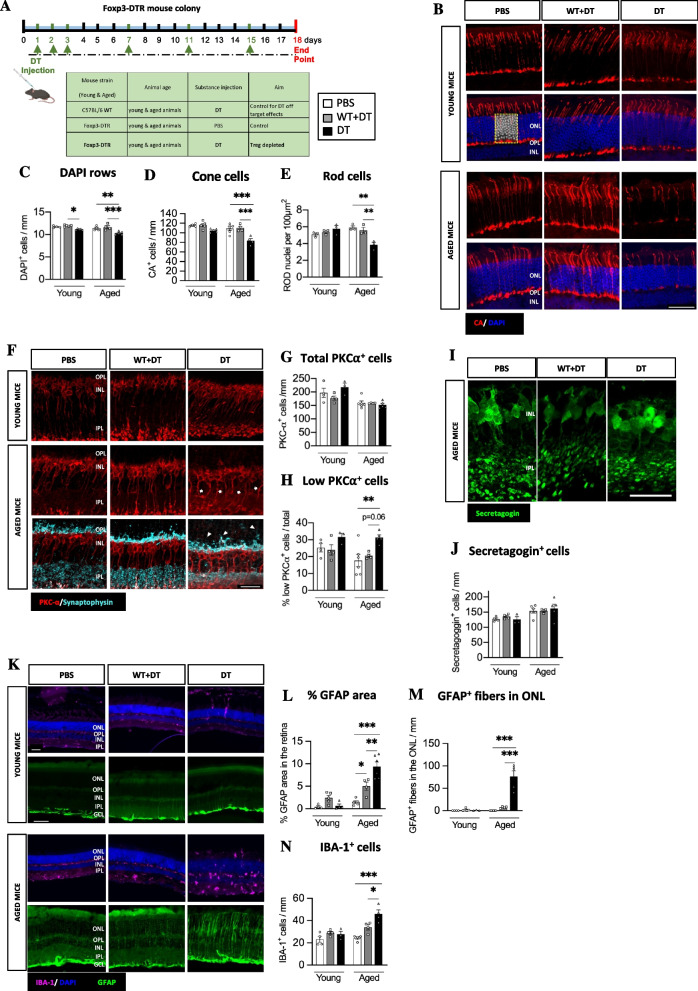
Treg depletion in aged but not young mice accelerates retinal neurodegeneration. **A** Diagram of the treatment regime and research groups. **B-E** Immunostaining and quantitative analysis of photoreceptors. Representative images showing photoreceptors in the ONL (**B**) (Cones, CA^+^; Rods DAPI^+^ CA^−^) in young and aged Foxp3-DTR (scale bar = 50 µm)**.** Quantitative analysis of the number of rows of DAPI^+^ nuclei in the ONL (**C**). Quantification of the number of cone photoreceptor cells (**D**) (CA^+^ cells) and the number of rod photoreceptors (**E**) (CA^−^DAPI^+^ cells in ONL). **F-J** Immunostaining and analysis of rod bipolar cells (**F–H**) and cone bipolar cells (**I, J**). Representative image of PKC-α (**F**) (red) and synaptophysin (blue, lower panels). Arrows indicate abnormal location of some PKC-α^+^ cell soma lower within the INL. Arrowheads indicate abnormal rod bipolar dendrite and synaptic vesicles sprouts into the ONL (scale bar = 25 µm). Quantitative analysis of the number of PKC-α^+^ cells (**G**) (*n* = 3–6 mice) and (**H**), percentage of PKC- α^+^ somas found in a lower location out of the total of PKC-α^+^ cells (*n* = 3–6 mice). Representative image (**I**) (scale bar = 25 µm) and quantification of rod bipolar cells in the INL (**J**) using secretagogin staining. **K-N** Analysis of IBA-1^+^ microglial cells and GFAP^+^ Müller cells determined by immunohistochemistry. Representative image of microglial cells (**K**) (IBA-1^+^, purple) and gliotic Müller cells (GFAP^+^ fibers, green) (scale bars = 50 µm). Quantitative analysis of the percentage of total GFAP^+^ area (**L**), the number of GFAP^+^ fibers in the ONL (**M**) and total number of IBA-1^+^ cells in all layers of the retina (**N**). Data information: B-N, *n* = 3-6 mice, data presented as mean ± s.e.m. *P < 0.05; ** *P* < 0.01; ***P < 0.001; 1-way ANOVA followed by Bonferroni's multiple comparisons test. Statistical comparisons that were not significant are not indicated in the graphs

In the healthy retina, Treg are present in the eye [13] and the retinal parenchyma [14]. Its presence, however, is notably sparse [13, 19]. To determine the number of Treg present in the aged eye, we performed flow cytometry staining of the retina and RPE/choroid of young (2 months) and aged (> 15 months) mice. As previously reported [13, 19], we found very few Treg in the retina of both, young and aged mice (~ 1–2 per mouse retina), which represented around 1% of the CD4^+^ T cells present (Additional Fig. 1 A, B). This number was increased in the RPE/choroid, were we found ~ 20 Treg cells per mouse and Foxp3^+^ Treg accounted for about 20% of the CD4^+^ T cells present (Additional Fig. 1C, D). However, we did not observe any difference in Treg number associated with age. To determine the role of Treg in the adult and aged retina in the absence of apparent disease, we depleted Treg using diphtheria toxin (DT) in young (< 4 months) and aged (> 18months) FoxP3-DTR mice [8, 20] (Fig. 1A). We first confirmed that Treg systemic depletion was successful characterising the presence of Foxp3^+^ Treg within CD4^+^ T cells and observed a significant Treg depletion in the lymph node, spleen and blood of both young and aged mice (Additional Fig. 1F-I). As previously described [20], we observed an enhanced systemic inflammation validated by the enhanced proportion of CD25^+^ CD4^+^ T cells in the lymph nodes (Additional Fig. 1J). Next, we evaluated changes in the neural retina and retinal pigment epithelium (RPE). Histological analysis at 2.5 weeks post-Treg depletion identified a significant decrease in total retinal thickness in aged but not young mice (Additional Fig. 2A, B). On further analysis, no changes in photoreceptor nuclei were observed in young mice lacking Treg, while aged mice had a significant reduction of total photoreceptors (DAPI^+^ rows in the ONL) following Treg depletion (Fig. 1B, C). Specifically, photoreceptor loss was associated with a decrease in both, Cone arrestin^+^ (CA^+^) cones (Fig. 1B, D) and rods, which were determined by subtracting the count of CA^+^ cells from the number of DAPI^+^ nuclei within a specific area (Rods, DAPI^+^CA^−^) (Fig. 1B, E). To further investigate Treg depletion-associated neurodegeneration, we examined the second-order neurons in the retina. In normal retinas, the bodies of rod bipolar cells reside in the outer region of the inner nuclear layer (INL). These cells exhibit a cluster of dendrites that extended into the outer plexiform layer (OPL). Treg depletion did not affect overall rod or cone bipolar cell number in young or aged Treg-depleted mice (Fig. 1F-J). However, in PKCα^+^ rod-bipolar cells and secretagogin^+^ cone-bipolar cells, Treg depletion in aged mice altered cell body laminarity (Fig. 1F, I). This was more pronounced in PKCα^+^ rod-bipolar cells; in which despite unaltered total number of somas, there was a significant increase of ectopic PKCα^+^ cell bodies located below the OPL due to shorter axons (Fig. 1F-H). Upon Treg depletion, we also found bipolar dendrites and synaptophysin sprouts expanding from the OPL towards the ONL (Fig. 1F). This cellular remodelling (consisting of the retraction of the bipolar cells, sprouting of dendrites and formation of ectopic synapses) has been previously observed in diseases associated with photoreceptor loss [21]. We did not observe any alteration in retinal ganglion cells across experimental groups (Additional Fig. 2C-E) suggesting that neurodegeneration following Treg depletion in aged mice predominantly impacted the outermost layers of the retina. Despite the overall systemic inflammation detected (Additional Fig. 1J), we did not observe an enhanced T cell infiltration in aged mice upon Treg depletion at the time point examined (Additional Fig. 2F-H), suggesting that the neurodegeneration observed in the aged Treg depleted mice is not linked to non-specific T cell activation and retinal infiltration due to the absence of Treg.

**Fig. 2:**
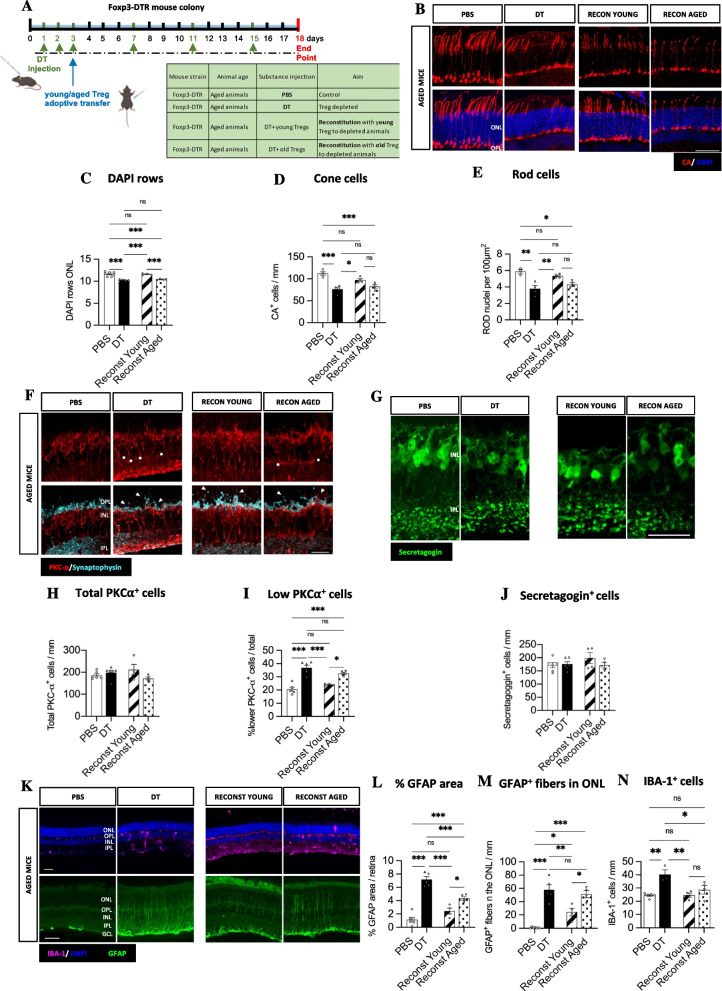
Adoptive transfer of young but not aged Treg rescues aged retinal neurodegeneration. **A** Diagram showing the experimental design. **B-E** Immunostaining and analysis of the effect of Treg adoptive transfer on photoreceptors. Representative images (**B**) (scale bar = 50 µm) and quantification photoreceptors (**C-E**). Total photoreceptors (**C**), CA^+^ cones (**D**) and CA^−^ rods (**E**) in the ONL of control, Treg depleted and Treg reconstituted aged retinas. **F-J** Alterations in bipolar cells after Treg adoptive transfer in aged animals. Representative images (**F**) (scale bar = 25 µm) and quantification of total rod bipolar cells (**H**) and rod bipolar cells with a shorter axon and displaced nuclei (**I**) identified by PKC-α (red) and synaptophysin (cyan) immunostaining (*n* = 4-6 mice). Representative images (**G**) (scale bar = 25 µm) and quantification of cone bipolar cells (**J**) identified by secretagogin immunostaining. **K-N** Changes in Müller cell gliosis and microglia in aged animals after the adoptive transfer of Treg. Representative image showing microglia (IBA-1^+^, purple) and Müller cell gliosis (GFAP^+^ fibers, green) (**K**) (Scale bar = 50 µm). Quantification of GFAP^+^ area (**L**), the number of GFAP^+^ fibers crossing the ONL (**M**) and total IBA-1^+^ microglia (**N**) in the aged retina. Data information: B-N, *n* = 3-7 mice, data presented as mean ± s.e.m. ns: not significant, *P < 0.05; ** *P* < 0.01; ***P < 0.001; 1-way ANOVA followed by Bonferroni's multiple comparisons test

### Retinal gliosis is associated with the lack of Treg in the aged retina

To determine if Treg depletion affected retinal glia, we first examined Müller glial cell responses. In the absence of Treg we observed a significant increase in GFAP^+^ staining in the neuroretina in aged but not young Treg-depleted mice (Fig. 1K), suggesting an intense Müller cell gliosis. In addition to overall increased GFAP expression (GFAP^+^ area of retina) (Fig. 1K, L) we also observed an expansion in reactive Müller cell number in the ONL (Fig. 1K, M). Müller cell hypertrophied branches could be a compensatory response growing towards the outer limiting membrane to compensate for photoreceptor loss in an attempt to maintain retinal cytoarchitecture, as described in other retinal diseases characterised by photoreceptor loss, such as retinitis pigmentosa, in which Müller cell hyperreactivity has also been observed upon photoreceptor loss [22].

We next examined whether Treg depletion modified microglial activity in the neuroretina. Microglia are the localized immune cells in the retina, and they show an increased activation in the aged retina [1, 3]. While Treg depletion in young mice did not affect retinal microglia, a significant increase in the density of IBA-1^+^ microglia was observed in the neuroretina of aged mice (Fig. 1K, N). This increase was detected in all retinal layers, but most significantly in the ONL which is normally devoid of microglia (Additional Fig. 3A). IBA-1^+^ cell increase was mostly related to ameboid-shaped microglia (Additional Fig. 3B), a morphological and phenotypic shift usually associated with pro-inflammatory responses [23]. This morphological change was further confirmed by measuring microglial branching as a function of the distance to cell soma. Treg depleted aged mice show a significantly decreased branching when compared to control aged mice (Additional Fig. 3C). To validate this pro-inflammatory phenotype, we quantified the number of MHCII^+^ pro-inflammatory microglia [24, 25] in aged Treg-depleted mice and observed a significant increase of these IBA-1^+^MHCII^+^ microglia in the neuroretina (Additional Fig. 3D, E).

**Fig. 3:**
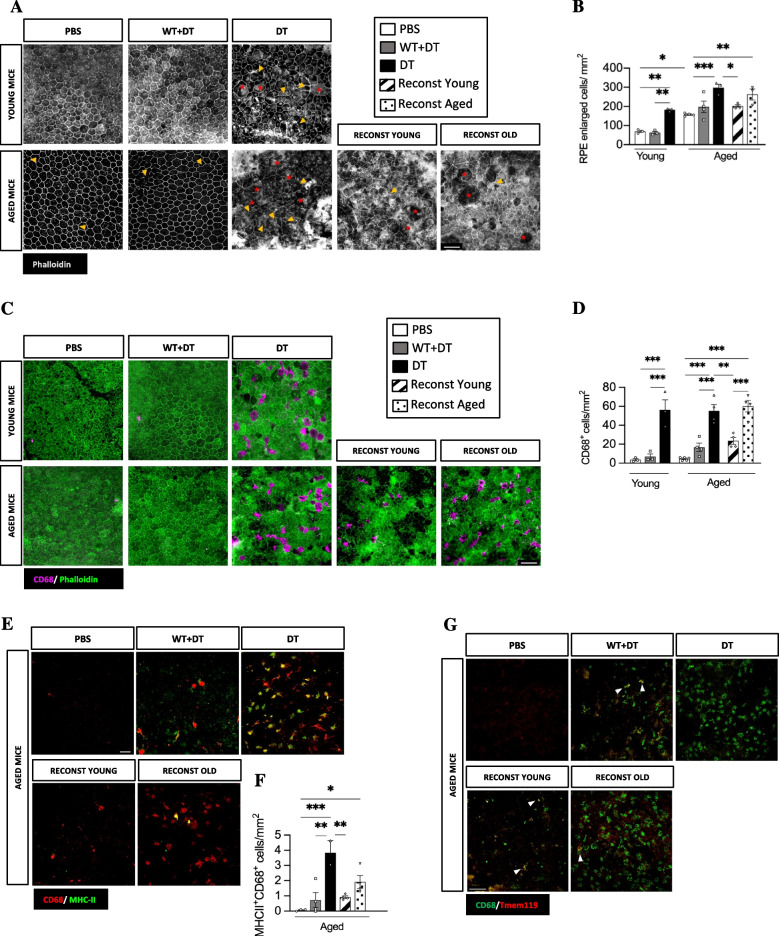
Treg depletion leads to blood-retinal barrier damage and innate immune cell infiltration in the subretinal space of young and aged mice. **A-B** Analysis of RPE cell changes. Representative image of phalloidin (**A**) indicating the geometry of RPE cells. Stars indicate enlarged cells and arrowheads fragmented cytoskeleton (scale bar = 50 µm). Quantification indicating the number of RPE cells with an enlarged morphology per area of RPE/choroid flatmount (**B**). **C-D** Determination of the number of CD68^+^ cells in subretinal space. Representative image of CD68 and phalloidin immunostaining in the RPE/choroid flatmount (**C**) (scale bar = 50 µm) and (**D**) quantitative analysis of the number of CD68^+^ cells in the subretinal space. **E–F** Determination of CD68^+^MHCII^+^ cells in the subretinal space. Representative image (**E**) (scale bar = 50 µm) and quantification (**F**) of CD68^+^MHCII^+^ innate immune cells in the subretinal space upon Treg depletion and reconstitution in aged mice (scale bar = 50 µm). **G** Confirmation of the CD68^+^Tmem119^−^ nature of the phagocytes observed in the subretinal space. Arrowheads indicate the scarce presence of CD68^+^Tmem119^+^ cells (scale bar = 100 µm). Data information: A-F, *n* = 2–5 mice, data presented as mean ± s.e.m. *P < 0.05; ** *P* < 0.01; ***P < 0.0051; 1-way ANOVA followed by Bonferroni's multiple comparisons test

These results indicate that Treg are necessary to prevent photoreceptor death and distorted lamination of bipolar cells in aged retinas. This appears to be linked with Müller cell hypertrophy as well as the accumulation of pro-inflammatory microglia in the nuclear layers of the retina.

### Treg adoptive transfer prevents retinal degeneration and rescues gliosis

We next sought to determine if Treg reconstitution could rescue age-associated retinal neurodegeneration. To do so, we adoptively transferred purified young and aged Treg (Additional Fig. 2I) into Treg-depleted aged mice to determine whether ageing affects the capacity of Treg to maintain retinal normal immunoregulation and limit the negative effects of an uncontrolled inflammation in the aged neuroretina (Fig. 2A). We first examined if reconstitution with young and aged Treg diminished photoreceptor loss. Surprisingly, transfer of young but not aged Treg rescued photoreceptor (cone and rod) loss in the neuroretina back to control levels (Fig. 2B-E). Although no differences were detected in the number of rod and cone bipolar cells upon Treg depletion (Fig. 2F, G, H, J), adoptive transfer of young but not aged Treg improved the bipolar lamination pattern, showing a decrease in the number of ectopic PKCα^+^ somas, as shown by the diminished number of PKCα^+^ cell bodies with a lower nuclear location (Fig. 2F, I). Additionally, young Treg also succeed in reducing the bipolar dendrite sprouting into the ONL (Fig. 2 F, I). Overall, young Treg adoptive transfer rescued retinal neurodegeneration features back to control levels, while aged Treg adoptive transfer was not significantly different to aged Treg depleted mice. We next evaluated if adoptive transfer of Treg in aged mice inhibited Müller cell gliosis. In line with what we observed for photoreceptor loss, only young but not aged Treg transfer prevented Müller cell gliosis in Treg-deficient aged retinas (Fig. 2K, L, M), supporting the concept of Müller cell gliosis being a compensatory response to retinal degeneration as described previously [26, 27]. Lastly, we studied the effect of Treg adoptive transfer on microglia density in the neuroretina. In contrast to what we observed with Müller cells, both young and aged Treg reduced the number of total IBA-1^+^ microglia as well as the number of IBA-1^+^MHCII^+^ microglia present in the neuroretina, however aged Treg were not capable of restoring microglial branching and complex morphology (Fig. 2K, N, Additional Fig. 3A-E). Therefore, since adoptive transfer of aged Treg reduces the number of microglia in the neuroretina, but not photoreceptor loss, these data indicate that the increased microglia in the neuroretina is not triggering photoreceptor loss and neurodegeneration at the outermost layers of the retina. The adoptive transfer of young Treg cells successfully rescued Treg depletion phenotype, as illustrated in Fig. 2. When the efficiency of adoptive transfer was assessed (in a separate study of the same animals aged Treg were not identified in blood, spleen or lymph nodes, in contrast to adoptively transferred young Treg [28]. Despite this observation, the fact that young Treg rescued retinal neurodegeneration phenotype (Fig. 2) and both, young and aged Treg rescued neuroretina microglial phenotype (Fig. 2K, N, Additional Fig. 2A, B, D, E) is evidence for the presence of adoptively transferred Treg cells in the organism. Despite adoptive transfer of young but not aged mice rescuing Treg depletion phenotype, we are cautious with the interpretation of these result, as not being able to detect adoptively transferred Treg in the classic lymphoid organs prevents us from determining whether the reconstitution levels were equivalent for both, young and aged Treg.

### Treg maintain RPE integrity and limit phagocyte accumulation in the subretinal space

Since the retinal neurodegeneration and gliosis observed in the absence of Treg in aged mice appears to be associated primarily with the outer retinal layers, which are in closer contact with the retinal pigment epithelium (RPE), we next evaluated the effect of Treg depletion in RPE integrity and immune cell infiltration in the subretinal space. It was previously reported that a range of RPE defects occur in the ageing retina. With age, RPE cells show an altered morphology, which includes discontinuity of the cytoskeletal bands between adjacent cells and altered cytoarchitecture (appearance of enlarged and irregular cells), contributing to altered retinal immune regulation and enhanced chronic low-grade inflammation described in the aged retina [29]. In agreement with this, we also observed significantly more enlarged RPE cells in aged animals (Fig. 3A, B). Next, we sought to investigate the effect of Treg depletion in age-related RPE alterations. Surprisingly, Treg depletion exacerbated age-related RPE pathology not only in aged, but also in young Treg depleted mice (Fig. 3A, B). In addition, CD68^+^ phagocytes were significantly increased in the subretinal space of both, young and aged mice upon Treg depletion (Fig. 3C, D). As per our observations in the neuroretina, phagocyte infiltration in the subretinal space mainly consisted of CD68^+^MHCII^+^ pro-inflammatory macrophages (Fig. 3E, F). Considering that RPE dysmorphology can lead to alterations in the outer blood retinal barrier, we next studied the nature of the phagocytes present in the subretinal space to determine whether they were mainly macrophages accumulated in response to systemic and/or local inflammatory cues, or alternatively, microglia that had migrated to the subretinal space because of the damaged RPE. Unlike what we described for the neuroretina, where the majority of IBA-1^+^ cells were Tmem119^+^ microglia (Additional Fig. 3F, G), in the subretinal space the vast majority of CD68^+^ cells were negative for Tmem119, indicating that CD68^+^ cells in the subretinal space are peripheral monocyte-derived macrophages, with few Tmem119^+^ microglia (Fig. 3G). Thus, Treg depletion led to accumulation of innate immune cells in the subretinal space and RPE alterations in both young and aged mice. However, these alterations were associated with neurodegeneration and gliosis only in the aged retina. Taking into account that neurodegeneration is present mostly in the outermost part of the retina, these data suggest that aged retinas are more susceptible to the neurotoxic effects of accumulated pro-inflammatory innate immune cells in the subretinal space. Hence, aged retinas could be more vulnerable and thus, have a higher dependency of Treg-mediated immunoregulation and maintenance of tissue homeostasis. Interestingly, transfer of young Treg diminished the RPE cell morphological changes, while transferred aged Treg failed to do so efficiently (Fig. 3A, B). Similarly, transfer of aged Treg did not limit CD68^+^ and CD68^+^MHCII^+^ cell infiltration in the subretinal space, while transfer of young Treg was highly efficient at doing so (Fig. 3C-G). In line with what was described in the retina, adoptive transfer of aged Treg did partially rescue the accumulation of pro-inflammatory MHCII^+^ macrophages in the subretinal space although to a lesser extent than young Treg, even though no statistically significant differences were detected between the two adoptive transferred groups (Fig. 3E, F). Therefore, Treg are essential to limit age-related RPE dysmorphology and innate cell accumulation in the subretinal space. However, older age Treg have a limited capacity to control an already established retinal inflammatory environment.

### Treg secretome rescues photoreceptor cell death upon inflammation ex vivo

The differential susceptibility of young and aged retina to Treg depletion might be related to a) a higher vulnerability of aged photoreceptors and RPE to systemic pro-inflammatory cues in the absence of Treg or b) differences in the local function of young and aged Treg in the retina. Considering that upon adoptive transfer young but not aged Treg can efficiently restore retinal neurodegeneration, we next investigated what changes in Treg with age.

**Fig. 4:**
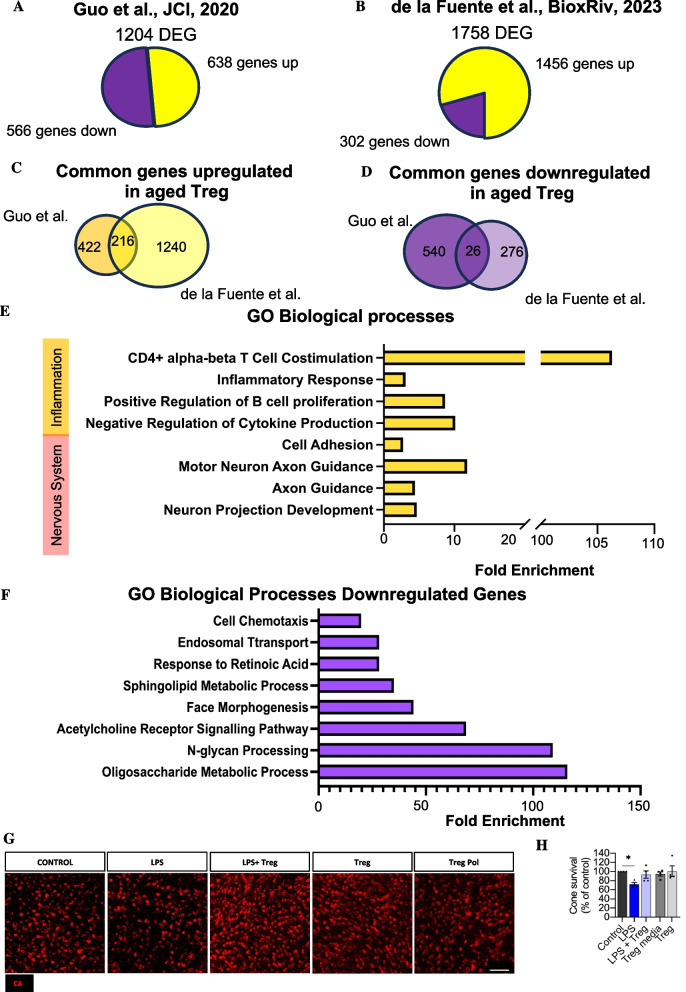
Identification of the biological processes enriched in aged Treg and effect of the secretome in retinal explants. **A-B** Differentially expressed genes (DEG) in aged Treg when compared to young Treg in Guo et al., 2020 (GSE130419) (**A**) and de la Fuente et al. 2023 (GSE218804) (**B**). **C, D** Venn diagram the genes that are commonly differentially expressed between both datasets. (**C**) Venn diagram showing the genes commonly upregulated in aged Treg across both datasets and (**D**) Venn diagram showing the genes downregulated in aged Treg in both datasets. **E** Quantification showing the fold enrichment of the GO Biological processes enriched amongst the genes upregulated in aged Treg and that are associated with inflammatory processes or nervous system processes. **F** Quantification showing the GO Biological processes fold enrichment of the genes downregulated in aged Treg. **G, H** Determination of the effect of aged Treg secretome in retina explants. (**G**) Representative images of cone-arrestin^+^ cones (CA^+^, red) in retinal explants 6h after treatment with LPS, LPS and Treg secretome, control Treg media and Treg secretome in vitro (Scale bar = 50µm) and (**H**) quantification of the cone survival shown as percentage of the control condition. Data information: E-F *n* = 4 mice, data presented as mean ± s.e.m. *P < 0.05; 1-way ANOVA followed by Bonferroni's multiple comparisons test

To do so, we took advantage of two available datasets investigating the transcriptomic changes associated with Treg ageing (Guo et al. 2020 (GSE130419); de la Fuente et al. 2023 (GSE218804)). Guo et al. found 1204 genes differentially expressed between young and aged Treg, with 638 genes upregulated and 566 genes downregulated with age (Fig. 4A), while in our dataset 1758 genes were found differentially expressed (DEG) with 1456 being upregulated and 302 downregulated in aged Treg (Fig. 4B). We focused on those genes that followed common traits for subsequent Gene Ontology (GO) analysis and combined both datasets, identifying 216 genes commonly upregulated (Fig. 4C) and 26 genes commonly downregulated (Fig. 4D). We next investigated the GO Biological processes associated to these genes and found that within the genes upregulated in aged Treg, GO Biological processes associated with inflammation (e. g. inflammatory response, regulation of cytokine production,) and the nervous system (e.g. axon guidance, neuron projection development) were enriched, suggesting that although Treg maintain most immune functions with age [30], aged Treg also acquire some pro-inflammatory phenotypes as shown by the increased expression of IFNγ and IL-1β or increased expression of other T cell effector genes [28, 31, 32] (Fig. 4E). The GO biological processes enriched in those genes that are downregulated with age are associated with sphingolipid metabolism, cell chemotaxis or acetyl choline receptor pathway (Fig. 4F).

Due to the systemic Treg depletion used in our diphtheria toxin model, it is not possible to determine whether Treg exert a local function within the retina or act indirectly through the maintenance of systemic homeostasis. To build on this and taking into account the enhanced inflammatory and nervous system related genes expressed by aged Treg, we next investigated local Treg effects in a retinal explant model [18]. We exposed retinal explants to Treg secretome in the presence or absence of a pro-inflammatory stimulus in the form of lipopolysaccharide (LPS) and investigated cone survival by staining for CA. LPS is a widely used pro-inflammatory activator of microglia and macrophages [33], which at least in part, will mimic the phenotype described in the aged retina when Treg are depleted. As described previously [34], LPS increased CA^+^ photoreceptor cell death, which was prevented by treatment with Treg-conditioned media (Fig. 4G, H). However, Treg conditioned media alone did not seem to enhance photoreceptor survival at least ex vivo*,* suggesting that Treg products limit immune-mediated retinal neurodegeneration. Nevertheless, the identification of the Treg secretome components that mediate this effect in the retina is outside the scope of this current work.

## Discussion

Even if the CNS has been for long-considered immune privileged, recent data has shown that Treg are also present in the healthy CNS, including the retina, albeit in very low numbers, suggesting that Treg have developed mechanisms that allow them to migrate through the blood brain and blood-retinal barriers [13, 14, 35]. Despite the paucity of Treg in the retina, in certain conditions, such as retinal neovascularization or uveitis, systemic Treg are actively recruited to the retina, where they influence microglial activation and attenuate disease severity [14, 15]. Ageing is characterized by a low-grade chronic inflammation, and a marked gliosis in different areas of the CNS, including the brain. Recent work showed that local expansion of Treg in the brain through interleukin-2 overexpression reverses molecular and cognitive signatures of ageing such as gliosis, inflammation, and cognitive decline [12]. Similar to what has already been described in the brain, disruption of retinal immune regulation is a key contributor to the development of age-related diseases [3]. The role of Treg has been extensively studied in the context of autoimmune and inflammatory eye diseases [36], but its role in retinal ageing and homeostasis in the absence of overt pathology remains unknown. In part due to the low number of Treg found in healthy retina, Treg have not been previously investigated in the context of the non-pathological ageing retina. Our study has addressed this question revealing a novel physiological role of Treg in maintaining retinal homeostasis and thereby limiting age-associated degenerative changes.

We have shown how Treg are essential in the maintenance of a healthy age-related para-inflammation, since its systemic depletion led to exacerbated neurodegeneration in the outermost layers of the neuroretina. Photoreceptor loss in the aged retina upon Treg depletion is accompanied by a marked glia cell response in the retina, with hypertrophy of Müller cells and increased microglial reactivity. Despite the significant changes observed in the ONL, we did not detect a change in ONL thickness associated with photoreceptor loss, probably compensated by Müller cell hypertrophy in the ONL [21]. Besides changes in the neuroretina, systemic Treg depletion is also associated with enhanced RPE dysmorphology and accumulation of macrophages in the subretinal space of both, young and aged mice. These alterations in the RPE, which is central in maintaining the integrity of the outer blood retinal barrier, could lead to the infiltration of pro-inflammatory phagocytes and additional pro-inflammatory factors that would exacerbate retinal neurodegeneration. However, we did not detect a significant infiltration of peripheral macrophages in the neuroretina as most IBA-1^+^ cells were also Tmem119^+^ and therefore, microglia. We also did not detect an enhanced infiltration of non-specific T cells in the young nor in the age retina in response to systemic inflammation, suggesting that RPE dysmorphology does not lead to an uncontrolled systemic immune cell infiltration in the neuroretina.

Although changes in RPE dysmorphology and the subretinal space upon Treg depletion are present irrespective of age, photoreceptors and retinal glial cells in young mice appear to be refractory to Treg depletion. This discrepancy between the effect of Treg in retinal homeostasis between young and aged mice may arise from the following factors: a) a pre-existing state of low-grade inflammation already present in the aged retina, which is further enhanced upon Treg depletion, b) enhanced vulnerability of aged photoreceptors which already show alterations that make them more susceptible to inflammation (e.g. increased lipid peroxidation, outer segment damage, reduced renewal capacity…) [37, 38], c) enhanced damage of the RPE with age which results in dysfunction related to the nourishment and protection of photoreceptors due to the accumulation of oxidative and nitrosative stress and accumulation of lipofuscin [29, 37, 39] and d) indirect damage of photoreceptors in response to Müller, astrocyte and microglia reactivity upon Treg depletion [6, 7], which is augmented in aged Treg depleted mice.

Additionally, we have shown that once immunoregulation in the ageing retina is lost, young Treg are more efficient at limiting inflammation and neurodegeneration than aged Treg, suggesting an intrinsic functional deficit in aged Treg in this setting. While young Treg are highly efficient fully restoring retinal homeostasis, aged Treg are only capable of limiting the presence of pro-inflammatory microglia and macrophages in the neuroretina and subretinal space but not they do not limit photoreceptor loss or gliosis. Despite the extent of the histological analysis performed, the absence of functional readouts such as electroretinography or visual acuity test, prevents us from determining neither the extent of functional impairment associated with Treg depletion, nor whether young Treg adoptive transfer translates into a full recovery of retinal functionality.

Young and aged Treg have a significantly different transcriptome [28, 31, 32], in which aged Treg show changes in genes associated with inflammation and the nervous system amongst other GO Biological processes. Although aged Treg can reduce the detrimental effect of LPS-mediated inflammation in retinal explants, it is plausible that the enhanced expression of inflammatory molecules in aged Treg reduces their capacity to limit inflammation in vivo as shown in the lung [32]. Here, we detected similarly low numbers of Treg in the young and aged retina and RPE/choroid, but it is possible that with age Treg have an altered migratory pattern with lower access to the retina or retinal proximity, similar to the results observed in muscle [40], impairing the capacity of adoptively transferred aged Treg to rescue retinal neurodegeneration. Despite retinal explant studies performed showing that aged Treg secretome can limit LPS-mediated photoreceptor cell death, the mechanisms by which Treg exert its functions in the retina are yet to be determined and warrant further investigation. One possibility to consider is that the retinal neurodegeneration observed is a consequence of the enhanced systemic inflammation developed when Treg are depleted due to the increased proliferation of effector T cells [20]. Whilst Treg depletion does not cause an overt T cell infiltration in the retina, at least at the time point examined, subsequent systemic inflammation may enhance the accumulation of phagocytes in the subretinal space in young and aged mice. Therefore, the augmented retinal neurodegeneration observed in the aged Treg depleted mice may be driven by the synergy between the enhanced systemic or local inflammation due to the absence of Treg and the extensively described age-associated low-grade chronic inflammation, which is already known to affect RPE integrity and increase photoreceptor vulnerability [37–39]. A limitation of this study is the inability to determine whether the role of Treg in maintaining retinal homeostasis is due to local Treg-mediated immune regulation or the maintenance of systemic immune homeostasis. Thus, further investigations focused on local Treg depletion via intravitreal DT injections as described in other models, are required [41].

Treg have key role in homeostasis maintenance due to their capacity to migrate to different tissues, including the retina or the CNS, irrespective of the presence of blood-retinal or blood–brain barriers and dampen inflammation, including the activation of other peripheral immune cells [20, 42] but also the activation of glial cells such as microglia, macrophages and astrocytes. This occurs through a range of mechanisms including the release of anti-inflammatory and neuroprotective factors, as we have shown with retinal explants, or alternatively through cell-to-cell contact [43, 44]. Several Treg-mediated molecular mechanisms have been shown to limit CNS-inflammation and foster neuroprotection in other CNS pathologies including, for example, osteopontin, amphiregulin, CCN3 or interleukin-10 [6–8, 45]. However, whether Treg directly signal to RPE cells, retinal glial and photoreceptors or alternatively, if Treg maintain systemic homeostasis to limit RPE dysmorphology and aged retinal neurodegeneration remains unknown. Thus, the precise molecular mechanisms underpinning the role of Treg in the homeostasis of the aged retina, and whether these are primarily local or systemic, warrants further investigation.

This study has identified Treg as key players in limiting age-associated retinal neurodegeneration, opening a new therapeutic avenue to preventing retinal degeneration in aged-related eye diseases. Future work that identifies the molecular mechanisms of Treg-mediated retinal neuroprotection could help to harness the anti-inflammatory and neuroprotective potential of Treg to treat age-associated retinal diseases.

## Supplementary Information

**Additional file 1. Fig. 1** Validation of Treg depletion and Additional Neuroretinal Characterization. **A-D** Flow cytometric characterization of Treg present in the young and aged retina and RPE/choroid. **A**, Gating strategy followed to identify Treg in the retina and RPE. (**B**, **C**) Flow cytometric plot showing retina (**B**) and RPE/choroid (**C**) expression of Foxp3 (y-axis) in concatenated CD4^+^T cells from young and aged animals. (**D**) Histogram showing the count of Foxp3^+^ Treg within CD4^+^ T cells in young (cyan) and aged (red) RPE/choroid. **E** Gating strategy followed to determine Treg depletion in lymphoid organs. F Flow cytometric plot identifying Treg by CD4, CD25 and Foxp3 staining. Analysis of endogenous Treg depletion quantified as the percentage of Foxp3 expressing CD4^+^ T cells in the lymph node (**G**), spleen (**H**) and blood (**I**) to verify Treg depletion. **J** Quantification of systemic inflammation by the expression of CD25^+^CD4^+^ T cells in lymph nodes of young and aged Treg depleted mice. Data information: **A-C** *n* = 4-5 mice, **D-G** *n* = 4-7 mice, H-I *n* = 4-7 mice. **E-I** data presented as mean ± s.e.m. *P < 0.05; ** *P* < 0.01; ***P < 0.001; U-Mann Whitney (E, F, G, H, I).

**Additional file 2.** **Fig. 2** Additional Neuroretinal Characterization. **A**, **B** Hematoxylin and eosin analysis of retinal thickness. Representative images showing hematoxylin and eosin staining (**A**) of young and aged retinas in the different treatment groups (scale bar = 50 µm). Quantification of the total retinal layer thickness as well as the thickness of the different cell layers (**B**). **C**-**E** Determination of RGC number in young and aged retinas. Representative image (**C**) (scale bar = 25 µm) and quantification of retinal ganglion cells identified by BRN3A^+^ (**D**) and DAPI^+^ staining in GCL (**E**). **F**-**H** Determination of T cell infiltration in the neuroretina of aged mice upon Treg depletion. Quantification of the presence of CD3^+^ T cells (**F**), CD3^+^ CD4^+^ T cells (**G**) and CD3^+^CD4^−^ T cells (**H**) per area of the neuroretina. **J** Quantification of the purity of the adoptively transferred Tregs shown as the percentage of CD4^+^ T cells expressing Foxp3. Data information: A, B *n* = 4-7 mice, C-E *n* = 3-6, F-H *n* = 2-7mice, J *n* = 4-5 isolations. B-J data presented as mean ± s.e.m. *P < 0.05; ** *P* < 0.01; ***P < 0.001; 1-way ANOVA followed by Bonferroni's multiple comparisons test (B-H), while U-Mann Whitney test was performed in J.

**Additional file 3.** **Fig. 3** Analysis of microglia morphology, distribution, and phenotype. **A** Quantification of IBA-1^+^ microglia in the different layers of the neuroretina. **B** Quantification of total, ramified, and ameboid microglia in the whole neuroretina of young and aged control and Treg depleted mice as well as aged-reconstituted mice. **C **Sholl analysis quantifying microglial branching as a function of the distance to the cell body. **D**, **E** Determination of IBA-1^+^MHCII^+^ cells in the neuroretina in aged mice. Representative image (**D**) (scale bar = 50 µm) and quantification of IBA-1^+^MHCII^+^ microglia in the neuroretina of aged control, Treg depleted and reconstituted mice (**E**). **F**, **G** Identification of IBA-1^+^Tmem119^+^ microglia in the neuroretina of young and aged mice. Representative image (**F**) (scale bar = 50µm) and quantification of IBA-1^+^Tmem119^+^ microglia in the neuroretina of young control, young Treg depleted, aged control, aged Treg depleted and aged reconstituted mice (**G**). Data information: A-B *n* = 3-6 mice, C *n* = 4-5 mice per group 10-20 cell per mouse analysed, D-E *n* = 4-7 mice, F-G *n* = 3-4 mice, data presented as mean ± s.e.m. *P < 0.05; ** *P* < 0.01; ***P < 0.001; 1-way ANOVA followed by Bonferroni's multiple comparisons test for all analysis except for C, which was analysed using two-way ANOVA.

## Data Availability

All original data is available from the corresponding authors upon reasonable request.

## Abbreviations

AMD: Age-Related Macular Degeneration
CA: Cone Arrestin
CNS: Central Nervous System
DT: Diphtheria Toxin
FoxP3-DTR: B6.129(Cg)-*Foxp3*^*tm3(Hebgf/GFP)Ayr*^/J
GFAP: Anti-Glial Fibrillary Acidic Protein antibody
IBA-1: Ionized Calcium Binding Adaptor Molecule 1
INL: Inner Nuclear Layer
i.p.: Intraperitoneal
OPL: Outer Plexiform Layer
PFA: Paraformaldehyde
PBS: Phosphate Buffered Saline
Treg: Regulatory T Cells
RPE: Retinal Pigment Epithelium
