# Supplementary figures and images for: Regulatory T cells limit age-associated retinal inflammation and neurodegeneration
Source: Mol Neurodegener. 2024 Apr 5;19:32. doi: 10.1186/s13024-024-00724-w (PMC10996107; doi:10.1186/s13024-024-00724-w)

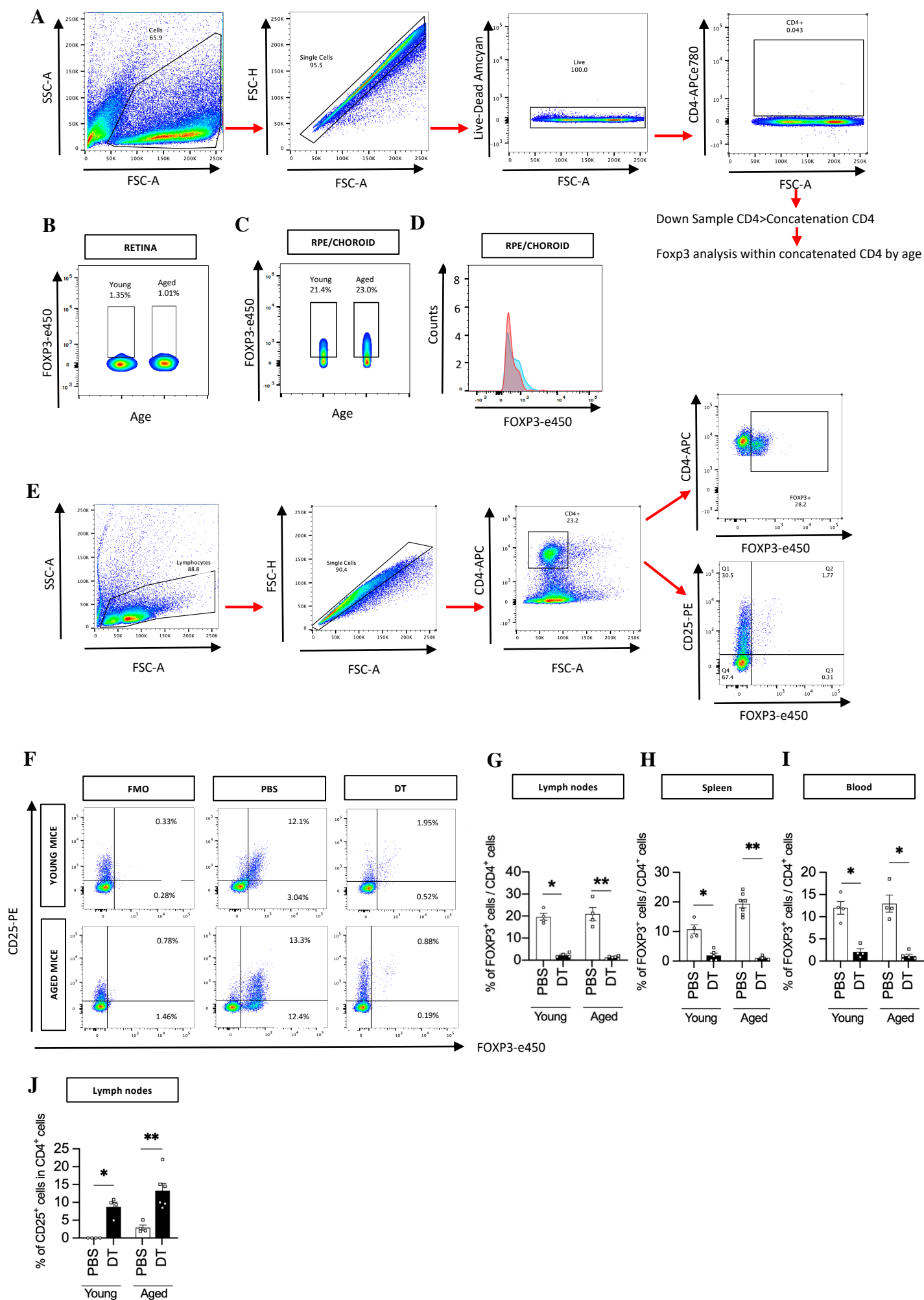

**Additional Fig. 1**

Supplement: Supplementary file 1 — Additional file 1. Fig. 1 Validation of Treg depletion and Additional Neuroretinal Characterization. A-D Flow cytometric characterization of Treg present in the young and aged retina and RPE/choroid. A, Gating strategy followed to identify Treg in the retina and RPE. (B, C) Flow cytometric plot showing retina (B) and RPE/choroid (C) expression of Foxp3 (y-axis) in concatenated CD4+T cells from young and aged animals. (D) Histogram showing the count of Foxp3+ Treg within CD4+ T cells in young (cyan) and aged (red) RPE/choroid. E Gating strategy followed to determine Treg depletion in lymphoid organs. F Flow cytometric plot identifying Treg by CD4, CD25 and Foxp3 staining. Analysis of endogenous Treg depletion quantified as the percentage of Foxp3 expressing CD4+ T cells in the lymph node (G), spleen (H) and blood (I) to verify Treg depletion. J Quantification of systemic inflammation by the expression of CD25+CD4+ T cells in lymph nodes of young and aged Treg depleted mice. Data information: A-C n = 4-5 mice, D-G n = 4-7 mice, H-I n = 4-7 mice. E-I data presented as mean ± s.e.m. *P < 0.05; ** P < 0.01; ***P < 0.001; U-Mann Whitney (E, F, G, H, I). [file 13024_2024_724_MOESM1_ESM.pdf]

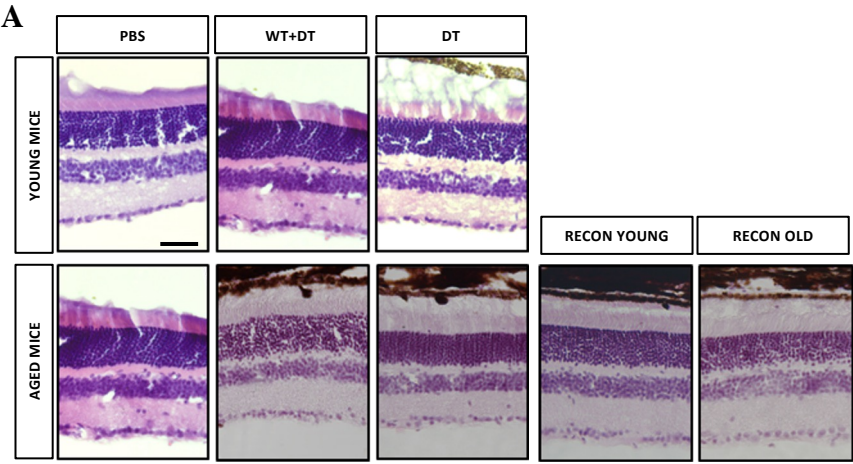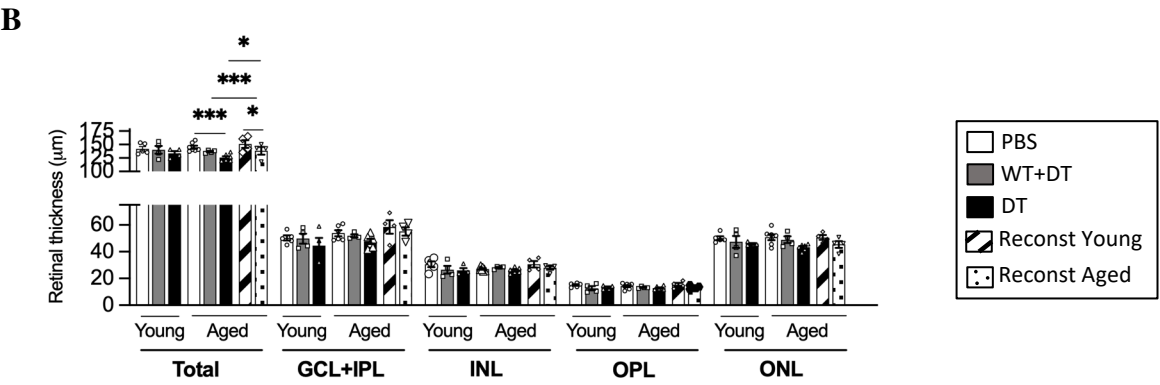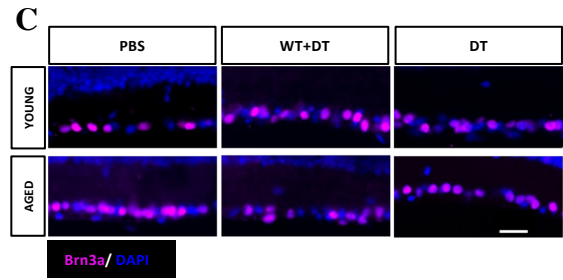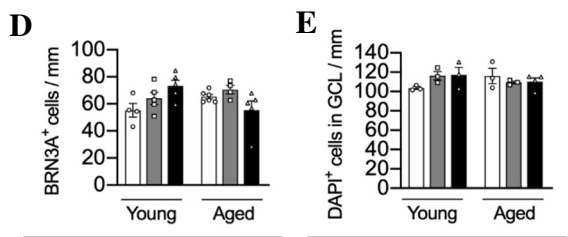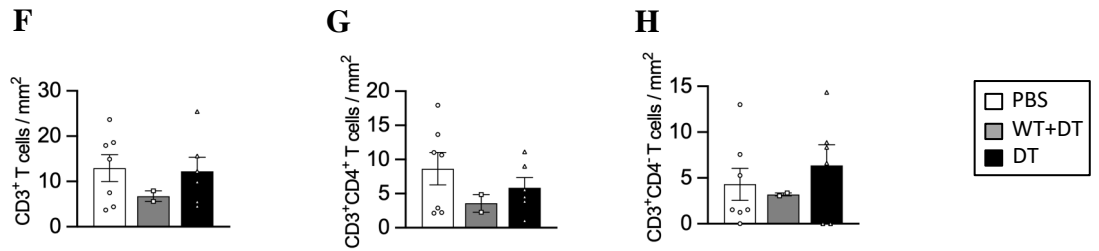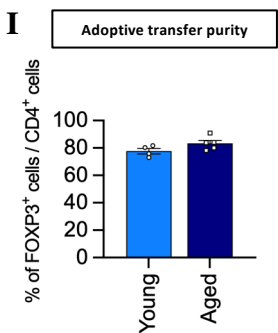

Supplement: Supplementary file 2 — Additional file 2. Fig. 2 Additional Neuroretinal Characterization. A, B Hematoxylin and eosin analysis of retinal thickness. Representative images showing hematoxylin and eosin staining (A) of young and aged retinas in the different treatment groups (scale bar = 50 µm). Quantification of the total retinal layer thickness as well as the thickness of the different cell layers (B). C-E Determination of RGC number in young and aged retinas. Representative image (C) (scale bar = 25 µm) and quantification of retinal ganglion cells identified by BRN3A+ (D) and DAPI+ staining in GCL (E). F-H Determination of T cell infiltration in the neuroretina of aged mice upon Treg depletion. Quantification of the presence of CD3+ T cells (F), CD3+ CD4+ T cells (G) and CD3+CD4− T cells (H) per area of the neuroretina. J Quantification of the purity of the adoptively transferred Tregs shown as the percentage of CD4+ T cells expressing Foxp3. Data information: A, B n = 4-7 mice, C-E n = 3-6, F-H n = 2-7mice, J n = 4-5 isolations. B-J data presented as mean ± s.e.m. *P < 0.05; ** P < 0.01; ***P < 0.001; 1-way ANOVA followed by Bonferroni's multiple comparisons test (B-H), while U-Mann Whitney test was performed in J. [file 13024_2024_724_MOESM2_ESM.pdf]

A

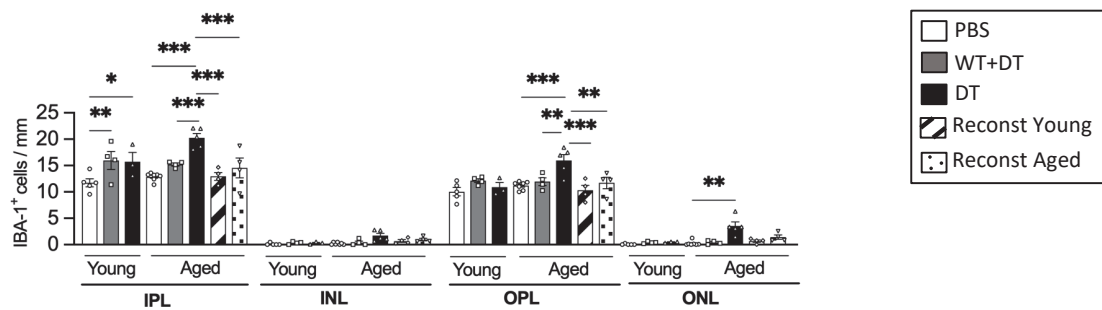

B

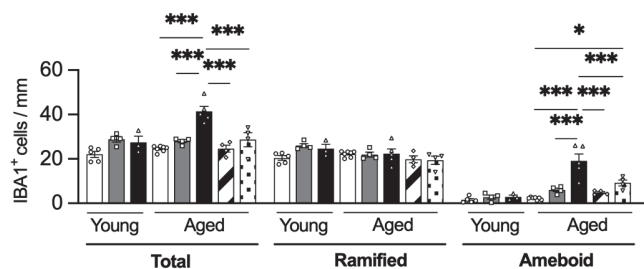

C

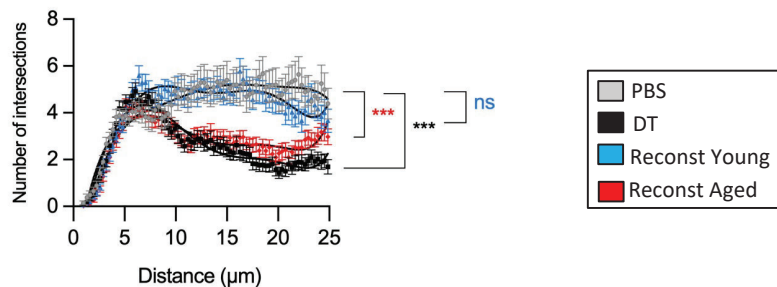

D

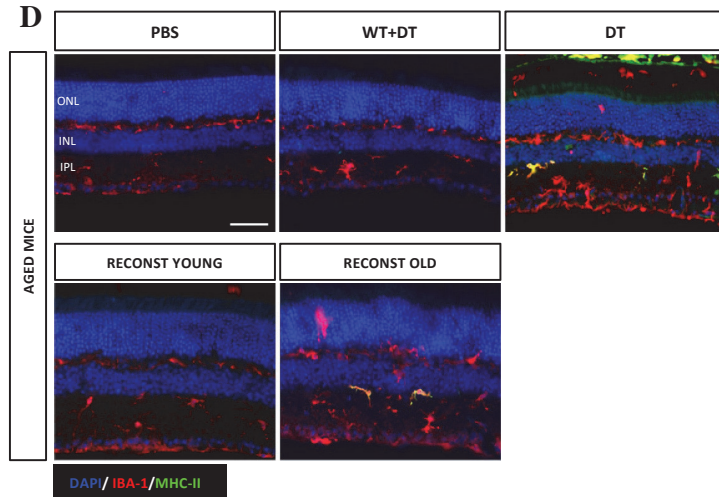

E

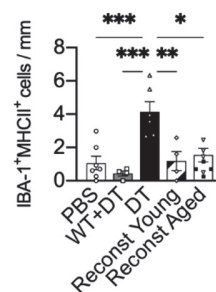

F

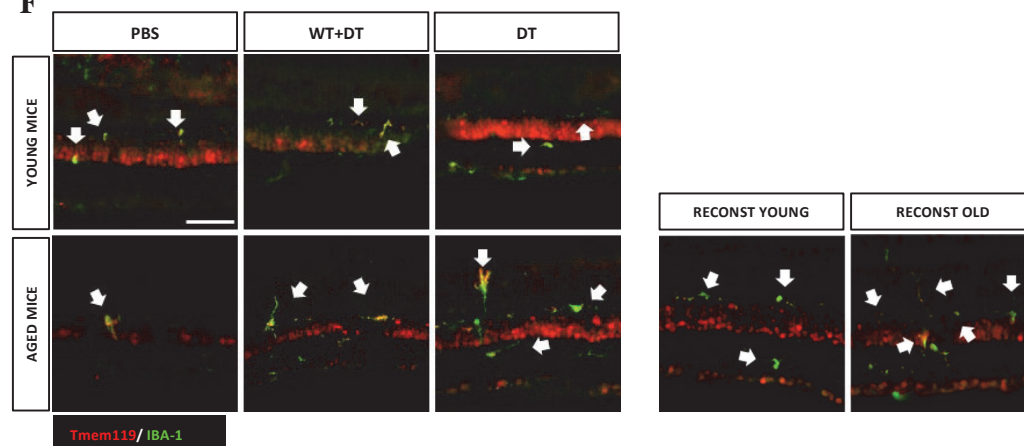

G

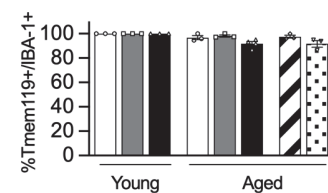

Supplement: Supplementary file 3 — Additional file 3. Fig. 3 Analysis of microglia morphology, distribution, and phenotype. A Quantification of IBA-1+ microglia in the different layers of the neuroretina. B Quantification of total, ramified, and ameboid microglia in the whole neuroretina of young and aged control and Treg depleted mice as well as aged-reconstituted mice. C Sholl analysis quantifying microglial branching as a function of the distance to the cell body. D, E Determination of IBA-1+MHCII+ cells in the neuroretina in aged mice. Representative image (D) (scale bar = 50 µm) and quantification of IBA-1+MHCII+ microglia in the neuroretina of aged control, Treg depleted and reconstituted mice (E). F, G Identification of IBA-1+Tmem119+ microglia in the neuroretina of young and aged mice. Representative image (F) (scale bar = 50µm) and quantification of IBA-1+Tmem119+ microglia in the neuroretina of young control, young Treg depleted, aged control, aged Treg depleted and aged reconstituted mice (G). Data information: A-B n = 3-6 mice, C n = 4-5 mice per group 10-20 cell per mouse analysed, D-E n = 4-7 mice, F-G n = 3-4 mice, data presented as mean ± s.e.m. *P < 0.05; ** P < 0.01; ***P < 0.001; 1-way ANOVA followed by Bonferroni's multiple comparisons test for all analysis except for C, which was analysed using two-way ANOVA. [file 13024_2024_724_MOESM3_ESM.pdf]
